# Efficacy and Safety of a New Formulation of Pancrelipase (Ultrase MT20) in the Treatment of Malabsorption in Exocrine Pancreatic Insufficiency in Cystic Fibrosis

**DOI:** 10.1155/2010/898193

**Published:** 2010-12-08

**Authors:** Michael W. Konstan, Theodore G. Liou, Steven D. Strausbaugh, Richard Ahrens, Jamshed F. Kanga, Gavin R. Graff, Kathryn Moffett, Susan L. Millard, Samya Z. Nasr, Édith Siméon, Jean Spénard, Josée Grondin

**Affiliations:** ^1^Cystic Fibrosis Center, Rainbow Babies & Children's Hospital and Case Western Reserve University School of Medicine, 11100 Euclid Avenue, Cleveland, OH 44106, USA; ^2^Cystic Fibrosis Center, The University of Utah, Salt Lake City, UT 84132, USA; ^3^Cystic Fibrosis Center, The University of Iowa Hospitals and Clinics, Iowa City, IA 52242, USA; ^4^Cystic Fibrosis Center, University of Kentucky, Lexington, KY 40536, USA; ^5^Cystic Fibrosis Center, Milton S. Hershey Medical Center and Pennsylvania State University, Hershey, PA 17033, USA; ^6^Cystic Fibrosis Center, West Virginia University, Morgantown, WV 26506, USA; ^7^Cystic Fibrosis Center, Helen DeVos Children's Hospital-Spectrum Health, Grand Rapids, MI 49503, USA; ^8^Cystic Fibrosis Center, University of Michigan Health System, Ann Arbor, MI 48109, USA; ^9^Department of Biostatistics, Quintiles Canada Inc., Ville Saint-Laurent, QC, Canada H4M 2P4; ^10^Clinical Development and Medical Affairs, Axcan Pharma Inc., Mont-Saint-Hilaire, QC, Canada J3H 6C4; ^11^Pharmacology Department, Université de Montréal, Montréal, QC, Canada H3C 3J7

## Abstract

*Background*. Pancreatic enzyme replacement therapy is the standard of care for treatment of malabsorption in patients with cystic fibrosis (CF) and exocrine pancreatic insufficiency (PI). *Aim*. To evaluate efficacy and safety of a new formulation of pancrelipase (Ultrase MT20) in patients with CF and PI. Coefficients of fat absorption (CFA%) and nitrogen absorption (CNA%) were the main efficacy parameters. Safety was evaluated by monitoring laboratory analyses, adverse events (AEs), and overall signs and symptoms. *Methods*. Patients (*n* = 31) were randomized in a crossover design comparing this pancrelipase with placebo during 2 inpatient evaluation periods (6-7 days each). Fat and protein/nitrogen ingestion and excretion were measured from food diaries and 72-hour stool collections. CFA% and CNA% were calculated for each period and compared. *Results*. Twenty-four patients provided analyzable data. This pancrelipase increased mean CFA% and CNA% (+34.7% and +25.7%, resp., *P* < .0001 for both), reduced stool frequency, and improved stool consistency compared with placebo. Placebo-treated patients reported more AEs, with gastrointestinal symptoms being the most frequently reported AE. *Conclusions*. This pancrelipase is a safe and effective treatment for malabsorption associated with exocrine PI in patients with CF.

## 1. Introduction

Exocrine pancreatic insufficiency (PI) is the most commonly reported gastrointestinal (GI) complication in patients with cystic fibrosis (CF), affecting approximately 90% of the CF population [1, 2]. PI results from obstruction of the pancreatic duct and leads to failure of pancreatic enzyme secretion (lipases, amylases, and proteases) [1–3]. If left untreated, PI results in the absorption of only 50% to 60% of dietary fats and proteins [4]. This malabsorption leads to depriving the body of energy it requires to maintain and promote growth. As a result, infants can experience failure to thrive, children and adolescents can have poor weight gain and/or growth failure, and adults can lose weight. In addition, the majority of patients with CF and PI have fat-soluble vitamin and essential fatty acid deficiencies [2, 5]. Evidence suggests that poor nutritional status may impair pulmonary function and may shorten the life expectancy of patients with CF [6, 7]. Other signs and symptoms of PI include abdominal distention and discomfort, flatulence, and frequent bulky, greasy, and foul-smelling stools, all of which may have a negative impact on the quality of life of affected patients.

While pancreatic enzyme replacement therapy (PERT) does not completely normalize PI, it does result in an increase in fat absorption above 85% in most patients with CF, and the advent of this therapy has contributed to a significant improvement in the outcomes of these patients [7–11]. Additionally, PERT allows patients with CF to eat a normal diet high in fat, absorb necessary nutrients, avoid many of the disabling GI symptoms associated with PI, and grow and develop more appropriately [1]. 

All currently marketed pancreatic enzyme products used to manage PI in patients with CF are a mixture of porcine-derived pancrelipase composed of varying amounts of lipase, amylase, and protease contained in an enteric-coated minitablet or microsphere [10, 12]. The enteric coating protects the enzymes from gastric acid-mediated degradation and is designed to dissolve at a pH above 5 to release proper amounts of enzymes in the upper small intestine where they digest fats, proteins, and carbohydrates [13]. Enteric-coated formulations have largely replaced nonenteric preparations for addressing PI in CF. 

The pancrelipase Ultrase MT (Axcan Pharma Inc., Mont-Saint-Hilaire, QC, Canada) consists of orally administered capsules containing enteric-coated minitablets of porcine-derived pancrelipase containing lipases, amylases, and proteases and is indicated for the treatment of patients with PI secondary to CF or other conditions [14]. The efficacy and safety of a former formulation of this pancrelipase (minitablets coated with Eudragit) has been demonstrated in 2 randomized, placebo-controlled studies performed in patients with CF and PI aged 7 years and older [8]. This pancrelipase was associated with a clinically significant increase (*P* < .0001) in the coefficient of fat absorption (CFA%) and nitrogen absorption (CNA%) compared with placebo. To increase the stability of the pancrelipase minitablets, the Eudragit coating was replaced by hydroxypropyl-methylcellulose phthalate-55 (HP55) in 2004, but the active pharmaceutical ingredients remain identical. The efficacy and safety of the HP55-coated formulation of this pancrelipase was assessed in this new randomized, placebo-controlled study in pediatric, adolescent, and adult patients with CF and PI.

## 2. Materials and Methods

### 2.1. Patients

Eligible patients included males and females aged 7 years and older with a confirmed diagnosis of CF and PI, the latter was confirmed by fecal elastase (FE-1) < 100 *μ*g/g of stool at the screening visit. With the exception of the underlying signs and symptoms associated with CF and PI, patients were to be clinically stable at study entry (based on medical and medication history, physical examination, and laboratory testing). Patients were taking optimal doses of a pancreatic enzyme product and were required to have adequate nutritional status (body mass index [BMI] ≥ 5th percentile for patients aged 7 to 20 years; and for those aged >20 years, BMI ≥ 16.0 kg/m^2^ for females and ≥16.5 kg/m^2^ for males). Patients were to be able to swallow capsules of study drug and to eat a high-fat diet (2 grams of fat/kg of body weight/day ± 15%). Women of childbearing potential were to have practiced an acceptable method of birth control for at least 1 month prior to study entry and to continue birth control for the duration of the study. Patients who used proton pump inhibitor (PPI) or histamine-2 blockers were allowed to continue their therapy during the study.

Exclusion criteria included known contraindication or hypersensitivity to Ultrase MT or to porcine proteins, allergy to the FD&C Blue no. 2 dye indicator (stool marker), use of narcotics, use of bowel stimulants or laxatives on a regular basis, use of any prohibited medication that could affect intestinal motility or absorption, history of bowel resection, or history of portal hypertension. Patients with acute pulmonary infection, acute pancreatitis, exacerbation of chronic pancreatitis, celiac disease, gastrointestinal dysmotility disorders, chronic or severe abdominal pain, poorly controlled diabetes, or with any conditions known to increase fecal fat loss were ineligible for the study. Patients with a current diagnosis or history of complete distal intestinal obstruction syndrome (DIOS) as defined by recent criteria [15] within the past 6 months or with 2 or more episodes within 1 year were also ineligible.

The study was conducted in accordance with the principles of Good Clinical Practice as identified by the International Conference on Harmonization and applicable national regulations [16]. The Institutional Review Board at each study site reviewed and approved the study protocol, consent and assent forms, and all related study documents. Written informed consent was obtained from all patients or their parents/legal representatives, and assent was also obtained from minors.

### 2.2. Study Design

This was a randomized, double-blind, crossover study that compared the efficacy and safety of this new formulation of pancrelipase (20,000 IU lipase/capsule) with that of placebo in the treatment of malabsorption in patients with CF and PI. The study was conducted at 8 centers within the United States between November 2006 and March 2007, and extensive and ongoing collaboration was maintained with the Cystic Fibrosis Foundation—Therapeutics Development Network Coordinating Center.

**Figure 1:**
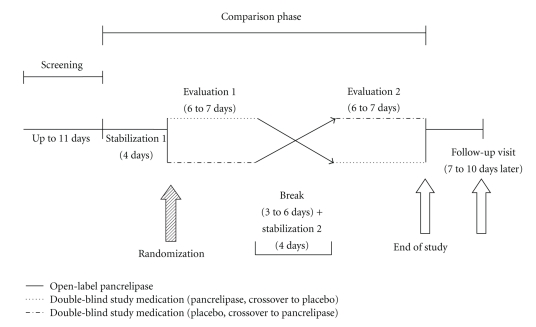
Schema of study design with a screening phase followed by a randomized crossover comparison phase that included 2 stabilization periods, a break period and 2 evaluation periods.

The duration of the study was from 41 to 49 days and included a screening phase of up to 11 days, a comparison phase of up to 28 days, and a follow-up visit (Figure 1). At the screening visit, patient eligibility was evaluated by detailed medical history, physical examination, and clinical laboratory assessments (hematology, chemistry, urinalysis, and serum pregnancy test for females of childbearing potential analyzed by Mayo Central Laboratory for Clinical Trials, Rochester, MN, USA, and FE-1 [by ScheBo test kit] analyzed by Genova Diagnostics, Asheville, NC, USA). 

A registered dietician at each study site developed a high-fat diet customized for each patient, which contained 2 g fat (± 15%)/kg body weight. This high-fat diet was to be introduced on the first day of the comparison phase and was continued throughout the course of the study, except during the break period. Patients received open-label pancrelipase during the entire screening phase of 11 days.

The screening phase was followed by the comparison phase, which consisted of 2 evaluation periods lasting 6 to 7 days each, separated by a break period. An outpatient stabilization period of 4 days on open-label pancrelipase preceded each inpatient evaluation period to establish similar baseline conditions. 

During stabilization periods 1 and 2, patients maintained their individualized high-fat diets and recorded the type and quantity of each food and liquid ingested for further assessment of fat, protein, and carbohydrate in their diet. The dose of pancrelipase (number of capsules) was adjusted and stabilized according to the clinician's observations and the patient's symptoms during stabilization period 1 to account for the increased amount of dietary fat. Patients were considered stable when they had 3 bowel movements/day or fewer, or when additional capsules of pancrelipase resulted in no further reduction in stool frequency. The stabilized dose (fixed number of capsules) was determined by the investigator at the end of stabilization period 1 and did not exceed 2500 IU of lipase/kg per meal or snack, as recommended in the CF Foundation Consensus Report on Nutrition for pediatric patients [17]. The stabilized dose was the dose of study drug dispensed during both inpatient evaluation periods as well as stabilization period 2.

Patients were admitted twice to a general clinical research center (GCRC) for 6 to 7 days to complete the evaluation periods. Randomization occurred on day 1 of evaluation period 1, and patients were administered the stabilized dose of the double-blinded study drug (pancrelipase or matching placebo) for the duration of the evaluation period. After a 3- to 6-day break and the second 4-day stabilization period, patients were readmitted to the GCRC to complete evaluation period 2 and crossed over to the opposite treatment received during evaluation period 1. 

Each patient's high-fat diet was carefully recorded by study personnel during both inpatient evaluation periods for further assessment of fat, protein, and carbohydrate. Study drug was administered under supervision with each meal or snack and recorded daily during both evaluation periods. The compliance to study drug was calculated by comparing the daily intake of capsules of study drug during inpatient evaluation periods with the stabilized dose established at stabilization period 1. Stools were collected as described below, and their frequency and characteristics were recorded in a diary during the collection period by study personnel. Patients had a physical examination including assessment of vital signs and laboratory evaluation at the end of each evaluation period. A follow-up visit was scheduled 7 to 10 days following discharge from evaluation period 2 or early discontinuation, for any patient who took at least 1 dose of study drug. Treatment-emergent adverse events (AEs) were assessed and recorded throughout the study using MedDRA codification, version 9.0 [18].

### 2.3. Stool Collections

The stool collections were performed in the GCRC and started on day 3 of each evaluation period. Patients were given 2 capsules containing 250 mg FD&C blue no. 2 dye (a stool marker) with their breakfast to mark the start of the stool collection. A second and similar dose of marker was administered 72 hours later (day 6) to mark the end of the stool collection, or exceptionally 96 hours later (day 7) if the first blue marker did not appear in the stool within 36 hours of administration. The first blue-tinted stool that appeared after administration of the first marker was not saved, but all stools (blue tinted or not) from subsequent bowel movements were collected and saved, up to and including the appearance of the first blue-tinted stool from the second marker administered on day 6 or 7. Stool samples from each evaluation period were sent to a central laboratory for analysis of cumulative fat and nitrogen content (Mayo Central Laboratory for Clinical Trials, Rochester, MN, USA). The diaries completed during stool collection by study personnel from all sites were analyzed by a registered dietician acting as a central reader, using a nutrition analysis software program (ESHA Food Processor Software, Salem, OR, USA) for fat and protein content. The CFA% and CNA% were calculated for each patient using the fat and nitrogen content of the stool and the fat and protein (converted in nitrogen equivalent) content of the food.

### 2.4. Statistical Analysis

The primary (CFA%) and secondary (CNA%) efficacy parameters of each treatment (pancrelipase and placebo) were compared using a semiparametric approach (Iman Conover). The entire set of observations was rank transformed, and a mixed model that included sequence, period, and treatment group as fixed effects and patient identification as a random effect was applied to the transformed data [19, 20]. Mean values were reported with their standard deviation (SD), and 2-sided statistical comparisons were carried out at the 0.05 significance level. The primary model was a main-effect model without interaction terms conducted in the primary efficacy patient population, which was defined to include all randomized subjects (intent-to-treat [ITT] population). Based on the results of a similar study and assuming a 2-sided *α*-error probability of 0.05 and a power of 80%, it was determined that a sample size of 24 evaluable patients was needed to be able to detect a minimum difference in CFA% of 18 with an SD of 30 between the 2 treatment groups. 

The per-protocol population included all patients who completed both evaluation periods without any major protocol violations and who provided efficacy data. The modified ITT (mITT) population consisted of all randomized patients who completed the 2 treatment periods and provided efficacy data (evaluable patients). All patients who received at least 1 dose of study drug, including open-label pancrelipase during the screening phase, were included in the safety population. Only descriptive statistics were performed on the safety data.

## 3. Results

### 3.1. Patient Disposition and Characteristics

Thirty-seven patients were screened, and 32 were enrolled in the comparison phase of the study. One enrolled patient started stabilization period 1 but withdrew consent and was not randomized to study treatment. The 31 randomized patients (ITT/safety population) ranged in age from 8 to 37 years with a mean ± SD of 19.6 ± 6.6 years. Five of the 31 patients discontinued the study prematurely: 1 for protocol deviation, 1 withdrew consent, and 3 for AEs. Twenty-six patients completed both study periods, but incomplete food recordings prevented calculation of CFA% and CNA% for 2 patients. Twenty-four patients provided complete efficacy data and comprised the mITT population. All 31 randomized patients were included in the safety analysis. All patients had severe pancreatic insufficiency as demonstrated by FE-1 < 100 *μ*g/g of stool. In the efficacy population, 62.5% of patients (*n* = 15) were using a PPI or histamine-2 blocker during the study, while 37.5% (9 patients) did not. Baseline characteristics of these patient populations are summarized in Table 1.

### 3.2. Diet, Treatment Exposure, and Compliance

The mean total daily dietary fat and protein intakes during the 2 double-blind evaluation periods were highly comparable in the mITT population (Table 2). Patients in the mITT population received a mean lipase dose of 6270 ± 2091 IU/kg/day while patients in the safety population received a mean lipase dose of 6062 ± 2049 IU/kg/day, with a range of 2830 to 10,619 IU lipase/kg/day. The mean lipase dose administered per meal or snack was 1262 ± 511 IU/kg with a range of 545 to 2292 IU/kg/meal or snack. For safety reasons, a consensus conference on the use of pancreatic enzyme supplements recommended that the daily dose of pancreatic enzymes for most patients should remain below 2500 U of lipase/kg per meal (10,000 U/kg/day) [21]. In the safety population, compliance in taking the study drug was high and consistent in the 2 evaluation periods. Average compliance was 98.3 ± 9.2% for the pancrelipase treatment period and 97.4 ± 7.6% for the placebo period.

### 3.3. Efficacy Assessments

Patients treated with pancrelipase had a significantly higher mean CFA% (88.6 ± 5.0%) than patients treated with placebo (53.9 ± 25.5%; *P* < .0001). The mean difference between treatment groups was 34.7 ± 25.0% (Table 2). Seventy-six percent of patients achieved a CFA% > 85% during pancrelipase treatment compared with 19% of placebo-treated patients, and nearly 50% of all patients achieved a CFA% > 90% during pancrelipase treatment. The absorption of proteins measured by CNA% showed similar results with a mean CNA% significantly greater (*P* < .0001) for the pancrelipase group (84.0 ± 7.4%) than for the placebo group (58.3 ± 20.6%) with a mean difference of 25.7 ± 17.7% between treatments (Table 2). The period and sequence effects for both CFA% and CNA% were not statistically significant (data not shown).

Patients treated with pancrelipase had 1.7 ± 0.6 bowel movements per day with 73.4 ± 27.6% described as being of normal stool consistency, compared with 2.9 ± 1.1 bowel movements per day with 31.6 ± 27.9% being of normal stool consistency during the placebo period (no statistical comparison performed).

### 3.4. Safety Assessments

Of the patients who received at least 1 dose of study drug, 17 (54.8%) and 27 (87.1%) reported treatment-emergent AEs during the pancrelipase and placebo periods, respectively. A total of 33 and 109 AEs were reported during the pancrelipase and placebo periods, respectively. No patient reported severe AEs while receiving pancrelipase compared with 5 patients (16.1%) receiving placebo who reported 7 severe AEs. Except for 1 severe AE (abdominal pain) judged as probably related to the placebo treatment by the investigator, the 6 other severe AEs were judged not related to treatment. There were 6 patients (19.1%) who reported at least 1 treatment-related AE on pancrelipase compared with 18 patients (58.1%) receiving placebo. The most frequently reported AEs were GI and consistent with CF, with fewer patients experiencing AEs of any type during treatment with pancrelipase than with placebo. No clinically significant effects of treatment were observed in any of the safety laboratory assessments, physical examinations, or vital signs.

## 4. Discussion

The primary end point of this study was the comparison of CFA%, calculated from all fat excreted in the stools over 72 hours and from concomitant fat intake, between the new formulation of pancrelipase and placebo. This measure is currently considered the gold standard to assess the efficacy of PERT in patients with CF and PI. The crossover design of this study, which uses patients as their own matched controls, allowed treatment differences between pancrelipase and placebo to be estimated with greater precision [22]. The baseline conditions were carefully reestablished before each evaluation period by the preceding 4-day stabilization periods and controlled for carryover effect of one treatment period to the other.

The treatment with pancrelipase significantly improved both fat (88.6 versus 53.9%) and nitrogen (84.0 versus 58.3%) absorption compared with placebo (*P* < .0001). The results are very similar to those obtained in a previous study performed with the former formulation of pancrelipase, where coefficients of fat and nitrogen absorption were 87.3 and 88.6%, respectively [8]. This suggests that the change in enteric coating did not affect the efficacy of this new formulation of pancrelipase. 

In the current study, a large majority of the patients reached a CFA% above 85% and almost half reached ≥90%. An increase in CFA% to ≥85% is considered a meaningful, significant improvement that approaches normal fat absorption [4, 23]. Other beneficial clinical aspects of pancrelipase treatment were observed in this study, including reduction in abdominal pain and frequency of stools, and a report of more normal stool consistency. Although the treatment period in this study was short, this pancrelipase seemed to be well tolerated, and no safety concerns were raised with this treatment in the population evaluated.

Excessive intake of exogenous pancreatic enzymes has been associated with the development of fibrosing colonopathy in patients with CF [24, 25]. Guidelines initially published in 1995 and updated in 2002 recommended no greater than 2500 IU of lipase per kg/meal, with a maximum daily dose of 10,000 IU of lipase per kg [17, 21]. In the current study, enzyme intake was considerably lower than these dose limits and well below doses associated with the development of colonic strictures.

## 5. Conclusion

Ultrase MT has been in clinical use for more than 10 years before the 2004 change in the enteric coating of the minitablets. The current study provides substantial evidence that this new formulation of this pancrelipase (HP55-coated) is both a clinically effective and safe pancreatic enzyme replacement therapy requiring relatively low doses for treating PI associated with CF.

## Figures and Tables

**Table 1:** Baseline characteristics of study patients.

	Safety population	mITT population
	*N* = 31	*N* = 24
Age (years)	19.6 ± 6.6	19.1 ± 5.9
Gender, *n* (%)		
Male	20 (64.5)	15 (62.5)
Female	11 (35.5)	9 (37.5)
Race, *n* (%)		
Caucasian	29 (93.5)	23 (95.8)
Black	2 (6.5)	1 (4.2)
Weight (kg)	55.6 ± 11.6	55.0 ± 9.6
BMI (kg/m^2^)	20.4 ± 2.2	20.1 ± 1.9
Fecal elastase-1, *n* (%)		
<15 (*μ*g/g of stool)	30 (96.8)	23 (95.8)
= 34 (*μ*g/g of stool)	1 (3.2)	1 (4.2)

Values are mean ± 1 standard deviation, unless otherwise noted. BMI: body mass index; mITT: modified Intent-To-Treat (patients completing both treatment periods).

**Table 2:** Dietary intake and absorption in the mITT population (*n* = 24).

	Treatment		Treatment
	Pancrelipase	Placebo	Difference*
Fat intake (g/day)	116.9 ± 21.9 (77–168)	119.8 ± 29.8 (69–219)	

Protein intake (g/day)	119.5 ± 39.6 (40–216)	120.0 ± 36.3 (40–188)	

CFA (%)	88.6 ± 5.0 (77–97)	53.9 ± 25.5 (14–97)	34.7 ± 25.0 (−7–75)

CNA (%)	84.0 ± 7.4 (62–95)	58.3 ± 20.6 (30–96)	25.7 ± 17.7 (−9–52)

All values expressed as mean ± 1 standard deviation (range).

*Mean treatment difference determined from within-patient differences.

CFA: coefficient of fat absorption; CAN: coefficient of nitrogen absorption; mITT: modified Intent-To-Treat (patients completing both treatment periods).
